# D-Limonene: A Promising Molecule with Bradycardic and Antiarrhythmic Potential

**DOI:** 10.5935/abc.20190233

**Published:** 2019-11

**Authors:** Quiara Lovatti Alves, Darizy Flávia Silva

**Affiliations:** 1 Laboratório de Fisiologia e Farmacologia Cardiovascular - Instituto de Ciências da Saúde - Universidade Federal da Bahia, Salvador, BA - Brazil

**Keywords:** Arrhythmia, Agents, Bradycardia, Monoterpenes, Limoneno, Monoterpenes, Medicinal Plants

It is unquestionable that cardiovascular disease (CVD) is and will continue to be one of the main health problems of modern society.^1^ A significant portion of cardiovascular disease-related deaths is a direct or indirect consequence of cardiac arrhythmias.^2^Cardiac arrhythmias are abnormal conditions that refer to any alteration in the normal sequence of cardiac electrical impulses, which can be classified according to frequency, mechanism, duration, and place of origin, resulting in inefficient pumping.^3^ More specifically, cardiac arrhythmias indicate alterations in the rate or regularity of the heart rhythm, as well as changes in the conduction sequence of physiological electrical impulses, from their origin in the sinoatrial node to their end, in the His-Purkinje system, and the ventricles.^4^The structural mechanisms that result in cardiac arrhythmias,^5^ as well as limitations regarding the safety and efficacy of “classic” antiarrhythmic drugs,^6^ make this disease difficult to treat.

The use of medicinal plants for the treatment, cure and prevention of diseases is one of the oldest forms of human medicinal practice.^7^ Natural medicinal plant-based products have shown to be an abundant source of biologically active compounds, many of which have been the basis for the development of new chemical products by the pharmaceutical industry.^8^Essential oils, extracted from aromatic plants, are natural products with a variety of biological actions, including cardiovascular properties.^9^Monoterpenes are the most representative molecules, which constitute 90% of essential oils,^10^ and also produce relevant effects on measurable cardiovascular parameters, including vasorelaxation and hypotension promotion in anesthetized and non-anesthetized rats.^11,12^ Additionally, monoterpenes may attenuate the development of hypertension in spontaneously hypertensive rats, by decreasing cardiac hypertrophy.^13^ Thus, it is evident that monoterpenes are molecules that could be further developed for the prevention and / or treatment of cardiovascular diseases. A study developed by our research group showed that the monoterpene carvacrol has negative chronotropic and inotropic effects on rats,^12^ whereas pulegone and geraniol have a negative inotropic effect on mammals.^13,14^

Nascimento et al.,^15^ in this issue of the *Arquivos Brasileiros de Cardiologia*, conducted an experimental study to evaluate the cardiovascular effects and potential antiarrhythmic response produced by the monoterpene D-limonene (DL) in rats. The study showed that DL produced intense and persistent bradycardia associated with hypotension in the heart of rats, corroborating the results observed in isolated hearts (*in vitro*), in which DL produced bradycardia and reduced left ventricular pressure. Additionally, the authors also observed antiarrhythmic activity using *in vivo* approaches.

The study is clear and provides evidence of the antiarrhythmic activity of DL. However, the mechanism of action involved in the responses to DL has not been studied and it is of the utmost importance to identify any potential differences with those drugs currently used in antiarrhythmic treatment. In addition, perhaps the use of an animal model of arrhythmia to evaluate the effects caused by subchronic treatment with DL could provide more information regarding the antiarrhythmic potential of this monoterpenoid. Clauss et al.,^16^ in an excellent review, described the various species currently used in arrhythmia research, including small and large animals.

Another unanswered question raised by this study was related to the probable DL action on Ca^2+^ channels. The authors suggested that the antiarrhythmic effect produced by DL may involve the inhibition of calcium channels.

Experiments demonstrating the effects of limonene on arrhythmias induced by Bayk8644 calcium channel activator are not sufficient, since any substance acting on different ion channels is relevant for the potential triggering of cardiac action, which could reduce the arrhythmias induced by Bay K, and not necessarily a consequence of the Ca^2+^ channel blocker. Thus, further studies are needed to clarify the exact mechanism by which DL promotes its antiarrhythmic effects.
